# Assessing local determinants of neural tube defects in the Heshun Region, Shanxi Province, China

**DOI:** 10.1186/1471-2458-10-52

**Published:** 2010-02-02

**Authors:** Jin-Feng Wang, Xin Liu, George Christakos, Yi-Lan Liao, Xue Gu, Xiao-Ying Zheng

**Affiliations:** 1 Institute of Geographic Sciences and Natural Resources Research, Chinese Academy of Sciences, Beijing 100101, China; 2 Department of Geography, San Diego State University, San Diego, California 92182-4493, USA; 3 Beijing Institute of Pediatrics, Beijing, 100012, China; 4 Institute of Population Science, Peking University, Beijing 100871, China

## Abstract

**Background:**

Neural tube defect (NTD) prevalence in northern China is among the highest worldwide. Dealing with the NTD situation is ranked as the number one task in China's scientific development plan in population and health field for the next decade. Physical and social environments account for much of the disease's occurrence. The environmental determinants and their effects on NTD vary across geographical regions, whereas factors that play a significant role in NTD occurrence may be buried by global statistics analysis to a pooled dataset over the entire study area. This study aims at identification of the local determinants of NTD across the study area and exploration of the epidemiological implications of the findings.

**Methods:**

NTD prevalence rate is represented in terms of the random field theory, and Rushton's circle method is used to stabilize NTD rate estimation across the geographical area of interest; NTD determinants are represented by their measurable proxy variables and the geographical weighted regression (GWR) technique is used to represent the spatial heterogeneity of the NTD determinants.

**Results:**

Informative maps of the NTD rates and the statistically significant proxy variables are generated and rigorously assessed in quantitative terms.

**Conclusions:**

The NTD determinants in the study area are investigated and interpreted on the basis of the maps of the proxy variables and the relationships between the proxy variables and the NTD determinants. No single determinant was found to dominate the NTD occurrence in the study area. Villages where NTD rates are significantly linked to environmental determinants are identified (some places are more closely linked to certain environmental factors than others). The results improve current understanding of NTD spread in China and provide valuable information for adequate disease intervention planning.

## Background

Birth defects --in particular, neural tube defects (NTD) - refer to any anomaly (functional or structural) that occurs in infancy or later in life. These are birth defects primarily of the brain and spinal cord (NTDs are comprised mainly of anencephaly, spina bifida, and encephalocele [1]). NTD is commonly induced by events preceding birth, inherited or acquired (usually between the 3rd and 4th weeks of the gestational age), and varying from minor cosmetic irregularities to life-threatening disorders. NTD are a leading cause of infant mortality and disability worldwide [2]. In rural areas of China, the cost for the health care system is high and is considered a prime poverty-causing factor in these areas.

Important birth defect factors include heredity, environment (physical and psychological conditions, socioeconomic status, health etc.) and their interactions. The impact of a risk factor varies with the type of NTD and the presence or absence of other defects. Several studies have investigated the role that genetic and environmental factors play in triggering NTD cases [3]. One of these factors is life-style. The psychosocial and emotional stress of the mother during pregnancy may increase NTD risk [4]. The views concerning the role of socioeconomic status in NTD risk vary, with some studies suggesting that the risk is higher among families of lower socioeconomic status, and some others not supporting this suggestion [5-7]. There are also racial and ethnic differences in NTD risk, which may be due to differences in genetic susceptibility, culture and diet [8]. Fortunately, many of the birth defect situations are preventable [9-11].

China has been acknowledged as a geographical region with high NTD occurrences. Based on data collected from a hospital-based surveillance system, the average NTD prevalence rate during 1987 was calculated to be approximately 27.4 per 10,000 births (with considerably higher rates observed in certain regions). Infant deaths caused by birth defects have increased constantly in China since the end of the last century [12]. Evidence obtained from urban and rural investigations clearly shows that since the 1990's the infant mortality rate (IFM) caused by birth defects in cities increased from 21 to 30% [13]. Accordingly, dealing with the NTD situation is ranked as the number one task in the country's scientific development plan for the next decade [14], a fact that amply demonstrates the high priority of NTD studies for China. The Shanxi province (north part of China) exhibits the highest rate of birth defects with 105.5 per 10,000 births in 1987; also, during the period 1996-2002 Shanxi had the highest rate with 60.88 per 10,000 births [15].

Taking into consideration the fact that the Heshun county (Shanxi province, China) is one of the areas with the highest NTD prevalence in the world, the main objective of this paper is to accurately map the geographical distribution of NTD cases in that county and identify the corresponding NTD determinants. Important issues to consider include the assessment of the specific factors causing NTD in a given region or population group in the Heshun county, and the investigation whether the NTD determinants apply globally or locally in space [16,17]. As far as the quantitative study of the disease is concerned, some of the relevant factors may be implicit in classical and global statistics analyses, whereas some others may possess a spatial dimension that needs to be determined by spatial statistics methods [18,19]. Furthermore, some NTD determinants may be leveraged out in a global statistics analysis (pooled dataset) but they may stand out in a localized analysis.

The above considerations make it appropriate that in the present Heshun NTD study we implement an adequate synthesis of quantitative techniques including spatial NTD analysis. The results of the spatial NTD analysis could be used to accurately identify intervention targets and offer valuable input to the systematic development of prevention strategies. This is an important matter, since it is widely accepted that the accurate identification of NTD determinants allows early intervention, which is a crucial component of any effort to minimize the consequences of birth defects.

## Methods

### Study area

**Figure 1:**
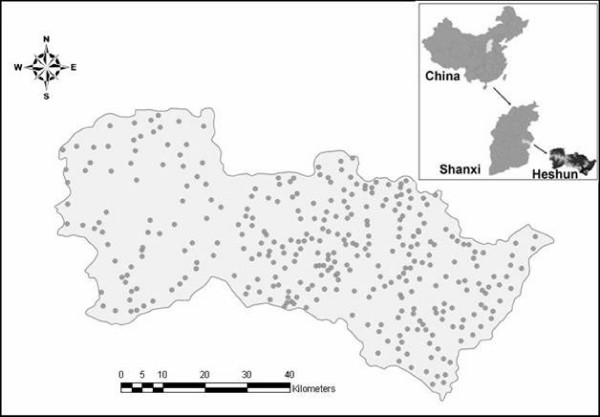
**Location of Heshun County (the dots on the map denote the 326 villages in which data were collected)**.

Heshun county (Fig. 1) is located at the Tai Hang mountain area of the Shanxi province and consists of 326 administrative villages with a total area of 2,250 km^2^. Most of the people in this county are farmers and their living environment seldom changes. There is no large-scale human immigration in the region's history. Remarkably, most kinds of birth defects designated by the WHO (World Health Organization) are found in Heshun, and among them the defects linked to NTD are the predominant ones [20]. Among the 7880 births in Heshun during 1998-2005, 187 of them suffered from NTD. The inherited and congenital causes of birth defects are similar among the region's population. Nevertheless, these causes explain only a small fraction of all NTD cases.

### Spatial random field theory

Let the geographical distribution of the epidemic attribute (number of NTD cases) be represented mathematically by the spatial random field (SRF), *Y*_**s**_, in the sense of Christakos [21]. The vector **s **= (*s*_1_, *s*_2_) denotes the location of the attribute, where *s*_1_, *s*_2 _are the associated spatial coordinates of the location. Also, let *m*_**s **_= *E *[*Y*_**s**_] be a non-random quantity that represents the average value of all possible SRF realizations at the location **s**, where the *E *[·] denotes stochastic expectation. The SRF formulation properly represents the fact that NTD prevalence shows significant variation in terms of geographic location (within regions and between countries [[5,22], and [23]]. Two quantitative expressions of prevalence rate can be defined in terms of the SRF, as follows.

The observed population rate (OPR) of the NTD cases *Y*_**s **_over an area ℜ is defined as

where **s **varies within ℜ, and |ℜ| denotes the total number of births in the area. The *r*_ℜ _is a random quantity, i.e. even when considering the same area, one may get different results if the *r*_ℜ _is computed over different SRF realizations. The superpopulation rate (SPR), also called the stochastic rate, of the NTD cases *Y*_**s **_over an area ℜ is defined as

where *f*_*y*, **s **_is the probability density function of *Y*_**s**_, and ψ_**s **_denotes a realization of *Y*_**s **_at s (for the underlying mathematical details the readers are referred to Christakos and Hristopulos [24]).

The OPR is directly observable and expresses the "here-and-now" crude disease rate, which makes *r*_ℜ _a useful study parameter when the objectives include the study of infectious disease outbreaks and the assessment of emergency health services. The SPR, on the other hand, expresses an essential property of epidemiological phenomena [25], which makes *m*_ℜ _a useful tool in the study of the relationship between an epidemic and its determinants. The SPR is rarely observed directly, but it can be approximated in terms of the available observations and by incorporating neighboring samples in a Bayesian context [26]. Accordingly, *m*_ℜ _will be the prime focus of the present study, which means that in the following the term "NTD rate" refers to SPR values.

### Prevalence rates

**Figure 2:**
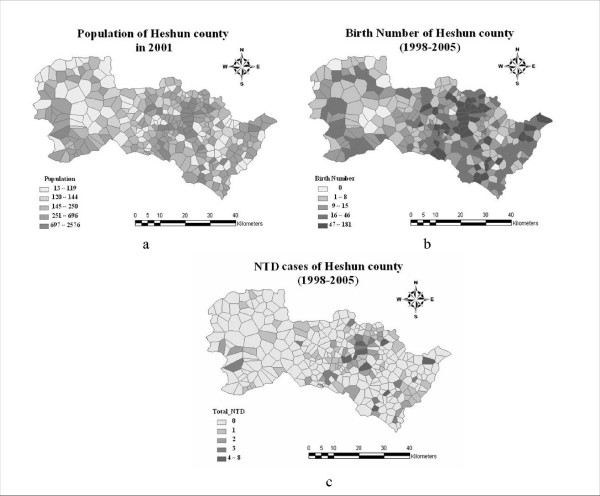
**Population and NTD cases in Heshun county**.

In order to investigate possible NTD determinants, the prevalence rates need to be estimated across space. In this study, determinants were considered at the village scale (Fig. 2a). The annual number of births, which is the denominator of the mathematical rate formula, was small and it varied highly at the annual scale (e.g., some villages had not a single birth in consecutive years). Since the NTDs constitute small probability events, the nominator is also expected to be small and highly variable annually. As a result, while the crude NTD rate exhibits high variability (simply due to the small sample size available), it does not reflect the essential attributes of the disease in a village. For example, for a village with a number of births equal to 2 and an NTD number equal to 1 during 2 years the calculated NTD rate is 0.5, which can not be the true risk of the disease, because the crude rate obtained from short-term observations within a small area does not reflect the true features of the long-term interaction between residents and negative environmental factors [26]. In addition to collecting data about NTD cases (Fig. 2c) and number of births (Fig. 2b) during as long time-periods as possible, one also needs to stabilize the estimated NTD rates, which is why the Rushton's circle method [27] was used in this study.

In spatial analysis one assumes that events are spatially correlated (dependent); see [27-29]. Accordingly, we introduced a regular lattice of grid points to serve as the spatial centers of the disease distribution. Then we constructed a series of circles that covered the entire Heshun area. Since the average NTD rates were calculated within each one of these circle using the available data, the circles were called "NTD_rate circle" (meaning that each circle had its own NTD rate). The socio-economic activities of Heshun residents usually take place between 6.2 and 9.3 Kms [20], which is why we set the radius of these circles to 3 Kms. Moreover, in order to cover all village points in the Heshun county, the least distance between any two centers should be 4.2 (3) Kms.

The Rushton circle [27] and the hierarchical Bayesian model [26] are efficient tools to correct the instability of the estimated rate for small probability events by borrowing strength from neighbors. Nevertheless, we still doubt that the number of unreliable values in the neighbors may play a more negative than positive role in estimation. Therefore, we set an artificial threshold of five live born of any village; villages bigger than this were included in the NTD prevalence rate calculation, then the values were used as sample in the subsequent statistical inference. Theoretically, there may exist a balance between (a) using larger samples to reduce the estimation variance [30,31], but at a risk of negative impact due to some highly unreliable values among the sample, and (b) using reliable but small samples in the estimation of a regional attribute. The matter deserves further investigation in the future.

**Figure 3:**
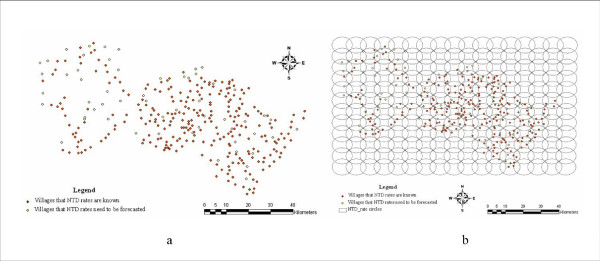
**Rushton circles to adjust NTD rates**. (a) Village centers; and (b) Village centers with "NTD_rate circles".

In this study, all 270 villages that have a number of births equal or higher than 5 ("≥ 5 " rule) were used to predict the "NTD_rate circle". Another 56 villages that did not satisfy the " ≥ 5 " rule were left to be predicted. The method used to predict the NTD rate across space involved the assumption that the villages had the same rate as the "NTD_rate circle" to which they belonged. It should be noticed that the "NTD_rate circles" may overlap each other when the distance between two centers is less than 6 Kms. Therefore, the villages to be estimated might not be included only in one circle, in which case the NTD rates of these villages were taken to be equal to the average values of the "NTD_rate circles" to which they belonged. Fig. 3a represents the village points of the Heshun county; solid points are the villages with reasonable NTD rates and hole points are those left to be adjusted. Prediction performance was assessed by means of the average root variance (ARV), in which case it was found that prediction improves when the distance between center points reduces (Fig. 3b). This finding was attributed to the fact that an increasing number of points may be included in some "NTD_rate circles" and, as a consequence, some of these points may be included in an increasing number of "NTD_rate circles". Interestingly, this effect did not increase significantly when the distance between the center points remained below 2 Kms. Therefore, we chose the 2 Kms as the distance between two center points for NTD rate prediction purposes; the corresponding ARV was 0.5347, which is an acceptable level reflecting the overall uncertainty of prevalence rate prediction (Fig. 3).

### Determinants of NTD and their proxies

Three prime types of factors are suspected to cause NTD: (1) environment (physical, social, economic etc.); (2) hereditary (genetic, pre-existing conditions etc.); and (3) synthetic (interaction between (1) and (2)). Recent studies show that most NTD cases are the result of environment-gene interactions [32,33]. The NTD etiology includes not only physical, chemical and biological agents, but also social and cultural determinants, where the latter affect body's immunity through their impact on psychological and mental health. Many of the direct NTD determinants are difficult or expensive to access. Actually, the direct etiological NTD factors act through at least four geographical layers, which are easier implementable in terms of a geographical information system, GIS [34,35]. In particular, these layers may be grouped are as follows:

• Physical NTD determinants that are spatially distributed. Potential NTD hazards include surface and subsurface water contaminated by insufficiently oxygenized ancient geological media; also, radiation emissions from certain rocks or along faults [36,37].

• Man-made pollution that is spatially distributed. Hazards of this kind include pesticides and chemical fertilizes spread over crop fields. Also, polluted air and water emission from workshops and electromagnetic radiation in the workplace [38-41].

• Nutrition processes that are spatially distributed. For example, nutrition strongly depends on spatially varying residential income. Hence, it is usually proportional to the GDP that is regularly surveyed across space and published in the government's annual statistics/census reports [42].

• Heredity and habits that are spatially distributed. Ethnic groups have specific genetically transmitted habits and behavioral patterns (e.g., related to food consumption), some of which are hazardous to health [43]. Health determinants may be detected when the disease cases and the ethnic characteristics share similar spatial patterns; for example, when the shape and size of spatial disease clusters are consistent with these of the citizens' daily activities, it could suggest that heredity is relevant to the regional NTD [20].

The explicit physical and human geographical proxies of the NTD determinants are collected: elevation, accessibility (e.g., road buffer), geological background (fault buffer), water conditions (e.g., river buffer), per-capita income (per-capita net income), medical conditions (e.g., number of doctors), crop yield (e.g., vegetable and fruit production), agricultural chemical exposure (fertilizer and pesticide use), land cover, lithology, watershed and soil conditions in every village. The socioeconomic factors were measured in terms of averaged annual levels during the period 1998-2005. Fumonisins in maze or other grains could be an important NTD factor [44,45]. However, the north of China where our pilot study was conducted is very dry during throughout the year, so the climate is not suitable for Fumonisins growth; and we have not found any report on Fumonisins in Shanxi province. In addition, the study area is hilly and is not the main maize production area, so we did not test for Fumonisins in this study.

### Inference approach

**Figure 4:**
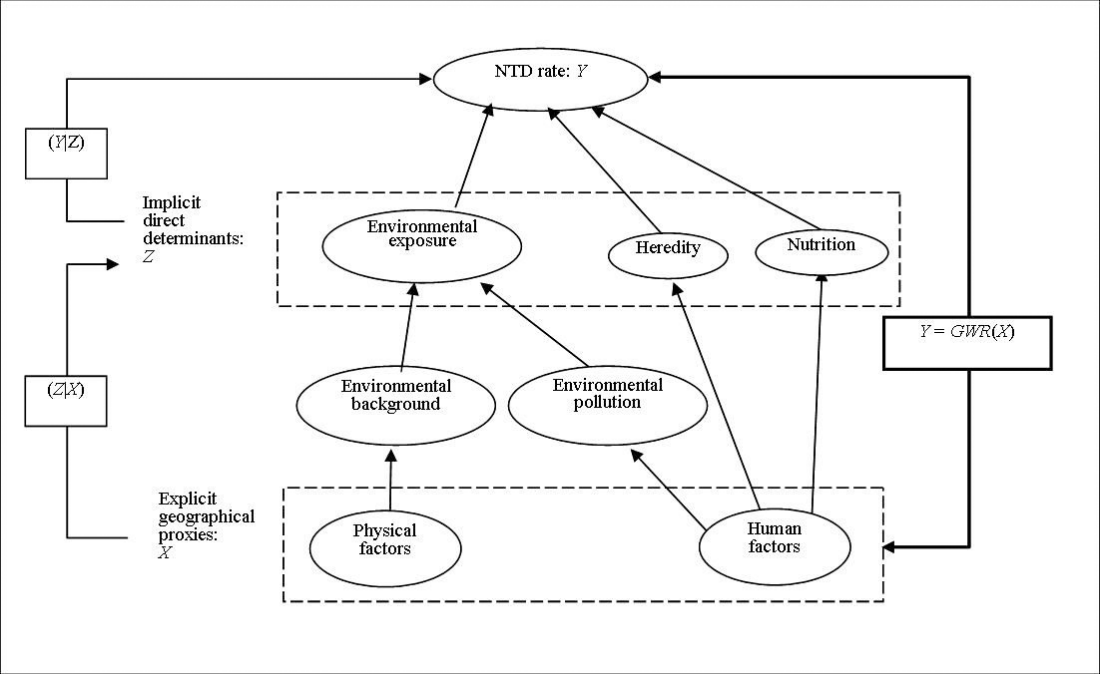
**Relationship between direct determinants and their proxy variables**.

A main objective of the present study is to identify possible NTD determinants. Fig. 4 illustrates a conceptual framework that involves the implicit direct NTD determinants *Z*_**s **_and their explicit geographical proxies *X*_**s**_. The latter are inserted into a GIS, which is then regressed with the NTD rate *Y*_**s **_by means of the GWR technique [46]. Finally, the results are interpreted in terms of the determinants *Z*_**s **_according to the conceptual framework of Fig. 4. Mathematically, the attributes *X*_**s**_, *Y*_**s **_and *Z*_**s **_are represented in terms of the SRF theory, see above. In symbolic terms, we seek to calculate the conditionals (*Z*_**s **_|*Y*_**s**_) and (*X*_**s **_| *Y*_**s**_), which logically infer the direct NTD determinant *Z*_**s **_given the NTD rate *Y*_**s **_and the proxies *X*_**s **_given the NTD rate *Y*_**s**_, respectively. In terms of Bayesian inference we can write [26]

where the (*Y*_**s**_|*X*_**s**_) is estimated by means of GWR, the (*X*_**s**_) is known from GIS, (*Y*_**s**_) is known from the corresponding survey, and (*Z*_**s**_| *X*_**s**_) is calculated on the basis of physical and human geographical processes. The Bayesian equations above allow logistic inference even when not all included variables and relationships are computable. In other words, the logical framework of Fig. 4 offers a valid means for the interpretation of NTD results in the Heshun county, quantitatively and qualitatively.

When implementing the GWR technique, categorical variables (including land cover, lithology, watershed and soil conditions) should be distinguished from explanatory variables. Typically, the former variables appear in terms of the Ordinary Least Squares (OLS) technique by introducing dummy variables. However, this would result in what is technically termed a "sever model design" in GWR analysis (i.e., an explanatory variable is perfectly collinear with the intercept, especially when a village and all its neighbors have the same values for one or more explanatory variables). As a consequence, we only regressed non-categorical variables and NTD rates using GWR. The categorical variables were checked by comparing the spatial patterns of the GWR outputs vs. those of the categorical variables.

### Local regression by GWR

In standard regression applications, the elastic coefficients of the NTD factors are assumed to be constant over space, which is the case of "one model fits all". The GWR technique, however, properly extends the traditional regression framework by allowing local rather than global parameters to be estimated. In this study, therefore, we implemented the GWR technique to identify local relationships between NTD and environmental factors. The GWR software tool used was GWR3.0 [47]; the ArcGIS9.0i was also used to map the variables and the numerical results obtained.

Note that the basic idea of GWR is that observations near a specified point **s**_*i *_have more influence in the prediction of the disease parameters associated with i than do observations farther away from **s**_*i*_. Accordingly, data close to **s**_*i *_are weighted more than data that are farther away from **s**_*i*_, in which case the geographical weights of observed data as far as prediction at point **s**_*i *_is concerned are as follows

where *w*_*ij *_represents the weight of any datum at point **s**_*j *_(*j *= 1, 2, ..., *n*) on the calibration of the prediction model at point **s**_*i*_. Normally, each *w*_*ij *_is a continuous function of *d*_*ij*_, the distance between **s**_*i *_and **s**_*j *_(i.e. *d*_*ij *_= |**s**_*i *_- **s**_*j*_|). One possible choice is , where *h *is called the bandwidth, and its specification depends on the situation under consideration.

The village centroids in the Heshun county are distributed unevenly: some are densely distributed, whereas some others are sparsely distributed. This means that local regression may rely on relatively few data points in areas where these points are sparsely distributed. To address this potential problem we used a spatially adaptive weighting technique, which involves the experimental calculation of the bandwidth rather than assigning it directly in the GWR context. The bandwidths are relatively small in areas where the data points are densely distributed and they are rather large in areas where the data points are sparely distributed. Better results, measured in terms of the global *R*^2 ^of GWR, were obtained when more points were involved in bandwidth calculation.

## Results

### Maps of GWR performance

**Figure 5:**
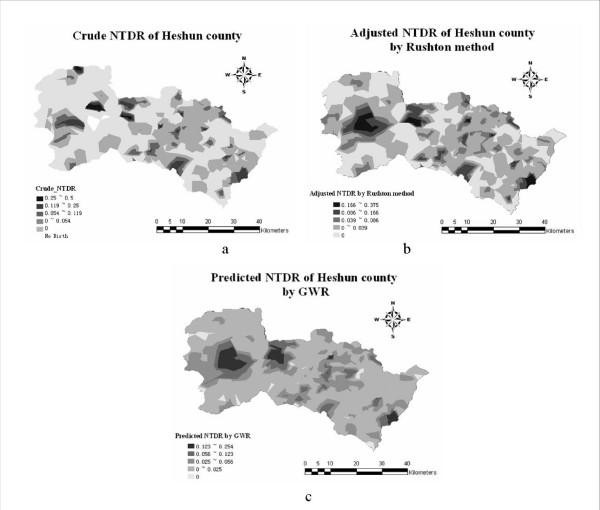
**NTD rates (NTDR) in Heshun County**.

**Figure 6:**
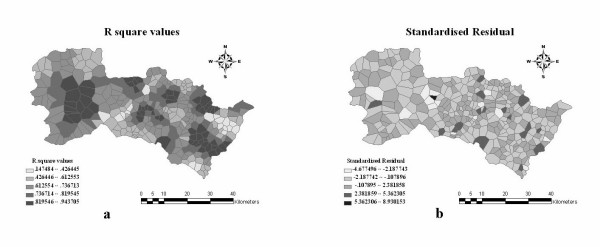
**Performance of the GWR technique**.

Fig. 5 illustrates the geographical distribution of NTD rates in crude form (a), adjusted by the Rushton method (b) and predicted by the GWR technique (c). The "geometric interval" is used in mapping cases in which the geometric coefficient can change once (in an inverse manner) in order to optimize the class ranges. The global performance of GWR is shown in Table 1 and the residual and *R*^2 ^maps of local regression are presented in Fig. 6.

**Table 1:** Global performance of GWR.

Parameter	P-value	
Intercept	0.00000	***

Elevation	0.00000	***

Riverbuffer	0.94000	n/s

Roadbuffer	0.00000	***

Faultbuffer	0.02000	*

Doctor	0.13000	n/s

Fertilizer	0.00000	***

Fruit	0.00000	***

Net_income	0.00000	***

Pestcide	0.00000	***

Vegetable	0.00000	***

*** = significant at higher than 1% level

* = significant at 5% level

### Maps of GWR coefficients

**Figure 7:**
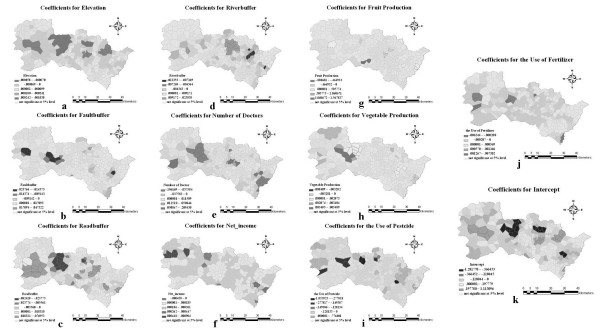
**Local GWR coefficients for every proxy variable together with the associate significant levels**.

The local coefficients for every variable (elevation, riverbuffer, number of doctors, net income, vegetable production, pesticide use etc.) together with the associated significant levels are plotted in Fig. 7. Different colors represent positive and negative coefficients, the significant levels are highlighted, whereas the insignificant values are masked in half transparency.

## Discussion and Conclusions

The series of maps in Figs. 7 ("NTD-proxies" links) together proxies and Fig. 4 (relationship between determinants and their proxies) provide a considerable amount of information concerning the relationship between NTD, its possible determinants and the proxies in the Heshun county (China). The maps accurately identify areas where NTD is sensitive to the various proxies; associate significant NTD determinants with the appropriate sites (using the inference framework of Fig. 4); and suggest potential sites for efficient intervention in order to effectively reduce the number of NTD cases.

### Nonlinearity and multi factors

The local GWR coefficients of most environment factors exhibit significant positive and negative associations with the NTD rates at different sites simultaneously. This cannot be explained by assuming a linear relationship between NTD and its possible determinants (which is the case, since GWR is essentially a linear regression technique). The situation may imply that a nonlinear association exists between NTD and the relevant environmental factors or that not a single determinant dominates the NTD occurrence in the study area.

### Elevation

NTD rates increase with increasing elevation in two village aggregations (the green color denotes significant positive association; Fig. 7a), whereas the rates decrease with increasing elevation in three other village aggregations (the purple color denotes significant negative association). All five places with significant coefficients of elevation are located in high NTD rate areas (Fig. 5c) --the reverse may not be true.

### Distance from faults

As is shown in Fig. 7b, in three village aggregates the NTD rates show significant negative association with distance from the faults, that is, the NTD reduces with increasing distance from the faults. Hence, the faults (in particular, the radiation and ancient water released from the faults) are the most probable significant NTD determinants in these three village aggregations.

### Distance from roads

The sites with significant road buffer coefficients (Fig. 7c) are consistent to those with high NTD rates (Fig. 5c). Furthermore, sites with significant positive coefficients (Fig. 7c) are located in the valley, whereas sites with negative coefficients (Fig. 7c) are found in the mountains. The situation in the valley is consistent with the common-sense expectation that people living near a road network usually have better life conditions than those living far away from it (which explains the lower NTD rates). Remarkably, the inverse situation is valid for people living in the mountains: the nearer to the road network they live, the higher the NTD rates. The epidemiological implication of the latter situation is complicated [48]. Distance from rivers. There is a relative large village cluster with significant positive river buffer coefficients (Fig. 7d), which may imply that in the case of the Heshun county the river plays a significant positive role in reducing the number of NTD cases. This role needs to be further investigated.

### Health services

Fig. 7e displays two purple-colored village aggregations, both in mountainous areas. In these areas, where health service is extremely rare, even a small improvement could lead to a significant reduction in NTD rates. These areas would be the most efficient places for NTD intervention (e.g., in terms of an increasing number of doctors), meaning that the highest intervention contribution in NTD reduction could be achieved in these areas.

### Net income

Fig. 7f shows that the net income distribution does not behave as is commonly expected --i.e. in relation to nutrition, thus becoming a significant determinant of NTD in the specific area. A possible interpretation is that in most villages of the Heshun county, the net income is not high or low enough to effect NTD rates; or the net income is not transformed into food or nutrition consumed by the local people. Note that three villages in the west county exhibit a highly positive association between NTD rates and net income (which probably means that confounding factors should be present in these areas).

### Fruit production

At its current level, fruit production (Fig. 7g) seems to be rather irrelevant to NTD. This does not imply that different levels of fruit production and the associated fruit consumption are irrelevant to NTD intervention, in general. Actually, fruit production and consumption in the Heshun county is very low, due to limitations imposed by the steep mountain and the barren soil in that county.

### Vegetable production

As one can see in Fig. 7h, there exists a village aggregation where NTD rates are positively associated with vegetable production. That is contrary to the common belief that the consumption of vegetables, which are rich in folio, will substantially reduce the risk of NTD. This is an abnormal village aggregation that deserves further investigation. Pesticides and fertilizers. The two factors (Fig. 7i and 7j) are believed to have negative impact on health [49]. There are several villages that present negative association between NTD rate and these two factors. This strongly suggests that the agriculture products (on which pesticides and fertilizers have been used) are not consumed locally (a common practice in rural China is that the local farmers use lower amounts of pesticide and chemical fertilizer for the products they plan to consume themselves).

### Baseline

The intercept (Fig. 7k) reflects the baseline of NTD rates, assuming that all variables in the equation are zero. Interestingly, the spatial pattern of significant intercept coefficients is consistent with that of road buffer (Fig. 7c) and elevation (Fig. 7a), but with an opposite sign in the case of elevation. Elevation and road affect human communication, which means that the above "NTD baseline-road-elevation" pattern consistency can play a significant role in NTD occurrences in the mountainous areas of the Heshun county. Intermarriage usually falls within the definition of social activities and the male and female usually have similar socioeconomic status when they get married [20].

### Soil and lithology

The categorical variables are visually compared with Fig. 7. The most of NTD cases happened in areas with soil type 7 (infant cinnamon soil). In contrast, the land cover types are distributed very dispersedly, so that one cannot detect any regular patterns. Also, the lithology of Q4 (Quaternary) is to some extent consistent with the negative coefficient for the fault buffer.

In summary, the local statistics approach identifies the villages where NTD rates are significantly linked to environmental determinants. In several village aggregations the NTD are found to be significantly associated with the proxy variables of radiation and ancient water released from the faults. Soil and lithlogy, river and road, health service, food production, pesticides and fertilizer are significantly related with NTD in some places, which can be interpreted by etiology or by social behaviors. Some places are more active than others as far as coefficient significance of the GWR is concerned, whereas most villages are always insignificant with respect to the different variables. This means that the NTD situation in these places may be more complicated than the variables and the linearity assumption considered by the GWR technique. In such cases, a composite space-time analysis involving nonlinear predictors [50-52] may be appropriate, which will be the subject of future NTD investigations.

## Abbreviations

NTD: neural tube birth defects; NTDR: neural tube birth defect rate; GWR: geographically weighted regression; SRF: spatial random field; OPR: observed population rate; SPR: superpopulation rate; ARV: average root variance; GIS: geographical information system.

## Competing interests

The authors declare that they have no competing interests.

## Authors' contributions

This study was conceived and completed by JW. XL and YL assisted with calculation. GC assisted with the analyses and revision of the manuscript. XG and XZ assisted with the medical analysis of birth defects. All authors read and approved the final manuscript.

## Pre-publication history

The pre-publication history for this paper can be accessed here:

http://www.biomedcentral.com/1471-2458/10/52/prepub
